# Supplementary material for: Prescription-based prediction of baseline mortality risk among older men
Source: PLoS One. 2020 Oct 29;15(10):e0241439. doi: 10.1371/journal.pone.0241439 (PMC7595371; doi:10.1371/journal.pone.0241439)
Supplement: S1 Table — The number of men having at least one mentioning of the code during the year preceding the index date, and the number of those that died, are provided. In PCBaSe prescriptions from all anatomical main groups A, B, C, D, G, H, J, L, M, N, P, R, S and V are available, except the pharmacological subgroups A01, A05, A09, A16 B02, B06 D01, D02, D03, D05, D06, D07, D08, D09, D10 G01, G02 J02, J04, J05, J06, J07 P01, P02, P03 R01, R05, V01, V03, V04, V07, and V08. (DOCX) [file pone.0241439.s001.docx]

**Table S1:** All ATC-codes at the level of pharmaceutical subgroups (having 4 positions in the code) observed in the study database. The number of men having at least one mentioning of the code during the year preceding the index date, and the number of those that died, are provided. In PCBaSe prescriptions from all anatomical main groups A, B, C, D, G, H, J, L, M, N, P, R, S and V are available, except the pharmacological subgroups A01, A05, A09, A16 B02, B06 D01, D02, D03, D05, D06, D07, D08, D09, D10 G01, G02 J02, J04, J05, J06, J07 P01, P02, P03 R01, R05, V01, V03, V04, V07, and V08.

| ATC-code | Number of men with ATC-code | Number of deaths |
| --- | --- | --- |
| A02A | 1420 | 879 |
| A02B | 49969 | 17392 |
| A03A | 3166 | 1251 |
| A03B | 335 | 116 |
| A03C | 3 | 2 |
| A03F | 2302 | 1296 |
| A04A | 908 | 610 |
| A06A | 30678 | 15259 |
| A07A | 1933 | 1040 |
| A07B | 5 | 2 |
| A07D | 3929 | 1803 |
| A07E | 2389 | 618 |
| A07F | 105 | 67 |
| A08A | 1071 | 252 |
| A10A | 17717 | 7868 |
| A10B | 30455 | 9829 |
| A11A | 202 | 131 |
| A11B | 6 | 4 |
| A11C | 2067 | 1256 |
| A11D | 244 | 130 |
| A11E | 9389 | 5515 |
| A11G | 171 | 87 |
| A11H | 644 | 282 |
| A11J | 138 | 73 |
| A12A | 9078 | 4383 |
| A12B | 5007 | 2829 |
| A12C | 979 | 469 |
| A14A | 13 | 5 |
| B01A | 115346 | 44200 |
| B03A | 7392 | 4592 |
| B03B | 31398 | 15102 |
| B03X | 903 | 697 |
| B05A | 1 | 1 |
| B05B | 142 | 111 |
| B05C | 297 | 253 |
| B05D | 72 | 59 |
| B05X | 49 | 37 |
| C01A | 7084 | 4401 |
| C01B | 1962 | 539 |
| C01C | 1034 | 289 |
| C01D | 27382 | 12320 |
| C01E | 25 | 11 |
| C02A | 298 | 103 |
| C02C | 3208 | 1098 |
| C02D | 106 | 54 |
| C02K | 22 | 12 |
| C03A | 18519 | 5519 |
| C03B | 156 | 138 |
| C03C | 33224 | 20579 |
| C03D | 9585 | 5530 |
| C03E | 9005 | 2866 |
| C04A | 90 | 38 |
| C05A | 4460 | 1223 |
| C05B | 1227 | 424 |
| C07A | 95791 | 33429 |
| C07F | 1276 | 346 |
| C08C | 55103 | 16620 |
| C08D | 3471 | 1511 |
| C09A | 62447 | 20799 |
| C09B | 9620 | 2175 |
| C09C | 30351 | 8722 |
| C09D | 14819 | 3232 |
| C09X | 4 | 2 |
| C10A | 92844 | 26312 |
| C10B | 41 | 3 |
| D04A | 349 | 124 |
| D11A | 1000 | 279 |
| G03A | 2 | 1 |
| G03B | 1429 | 295 |
| G03C | 15 | 3 |
| G03D | 3 | 0 |
| G03F | 2 | 1 |
| G03G | 10 | 2 |
| G03H | 27 | 19 |
| G03X | 5 | 3 |
| G04B | 24377 | 5293 |
| G04C | 28784 | 9642 |
| H01A | 142 | 34 |
| H01B | 507 | 196 |
| H01C | 90 | 48 |
| H02A | 20259 | 7965 |
| H03A | 9913 | 3493 |
| H03B | 155 | 64 |
| H04A | 184 | 74 |
| H05A | 8 | 2 |
| H05B | 177 | 122 |
| J01A | 17156 | 5158 |
| J01C | 45776 | 14289 |
| J01D | 3201 | 1429 |
| J01E | 5348 | 2612 |
| J01F | 6423 | 2231 |
| J01G | 9 | 4 |
| J01M | 15625 | 6291 |
| J01X | 2579 | 1404 |
| L01A | 326 | 277 |
| L01B | 600 | 253 |
| L01C | 49 | 43 |
| L01D | 11 | 5 |
| L01X | 579 | 345 |
| L02B | 71 | 19 |
| L03A | 401 | 188 |
| L04A | 4358 | 1352 |
| M01A | 53664 | 11432 |
| M01C | 27 | 11 |
| M02A | 4642 | 1768 |
| M03A | 66 | 17 |
| M03B | 3217 | 966 |
| M03C | 1 | 0 |
| M04A | 12264 | 5576 |
| M05B | 3082 | 1476 |
| M09A | 371 | 81 |
| N01A | 2 | 1 |
| N01B | 1696 | 1004 |
| N02A | 37857 | 14021 |
| N02B | 52772 | 22022 |
| N02C | 1832 | 457 |
| N03A | 9439 | 4205 |
| N04A | 728 | 347 |
| N04B | 5396 | 2940 |
| N05A | 6713 | 3810 |
| N05B | 22787 | 10237 |
| N05C | 40238 | 17257 |
| N06A | 28273 | 12236 |
| N06B | 299 | 75 |
| N06D | 4025 | 3258 |
| N07A | 159 | 54 |
| N07B | 2964 | 936 |
| N07C | 67 | 8 |
| N07X | 65 | 52 |
| R02A | 19 | 7 |
| R03A | 23123 | 8519 |
| R03B | 16999 | 7081 |
| R03C | 459 | 251 |
| R03D | 1554 | 638 |
| R06A | 14765 | 4241 |
| R07A | 7 | 6 |
| S01A | 10060 | 3237 |
| S01B | 10419 | 3778 |
| S01C | 213 | 53 |
| S01E | 14498 | 5774 |
| S01F | 1021 | 289 |
| S01G | 3299 | 764 |
| S01H | 22 | 2 |
| S01L | 4 | 2 |
| S01X | 5626 | 2604 |
| S02A | 461 | 129 |
| S02B | 1301 | 265 |
| S02C | 1138 | 251 |
| S02D | 1 | 0 |
| S03C | 9676 | 2734 |
| V06D | 74 | 67 |
