# Supplementary figures and images for: Prescription-based prediction of baseline mortality risk among older men
Source: PLoS One. 2020 Oct 29;15(10):e0241439. doi: 10.1371/journal.pone.0241439 (PMC7595371; doi:10.1371/journal.pone.0241439)

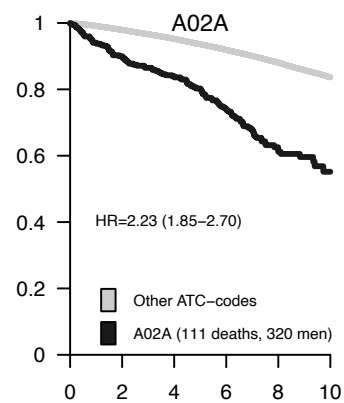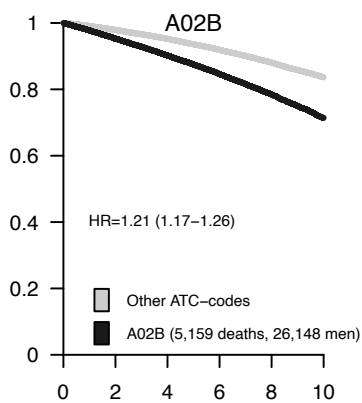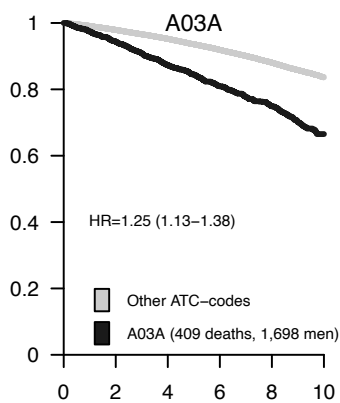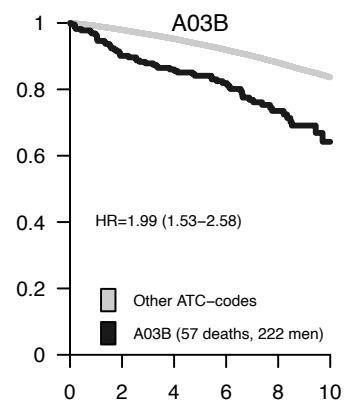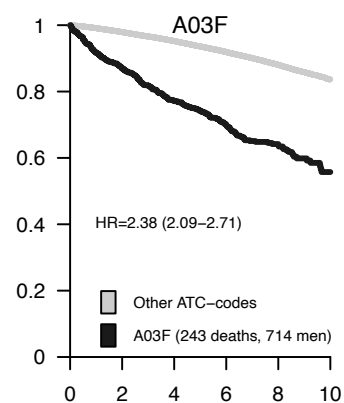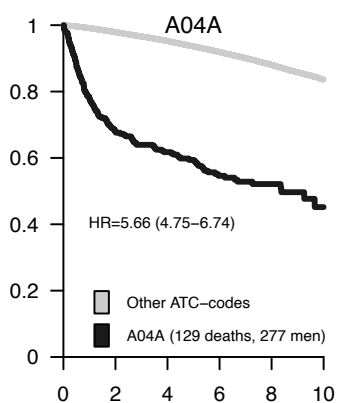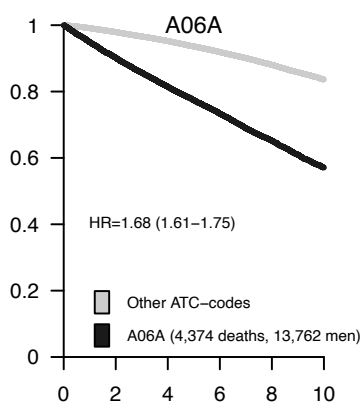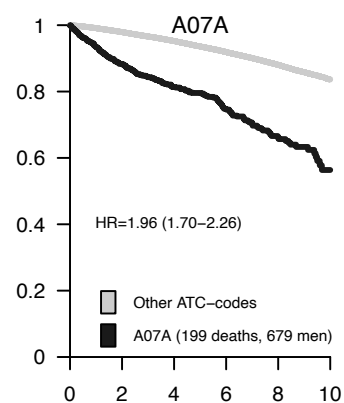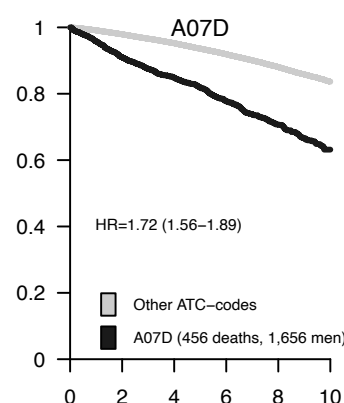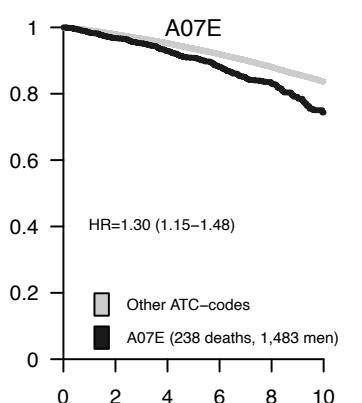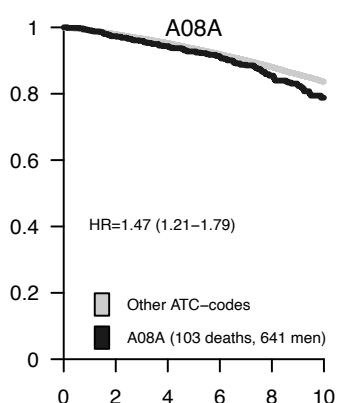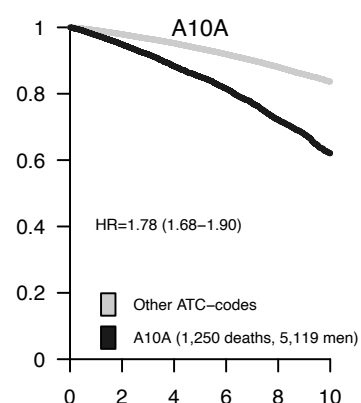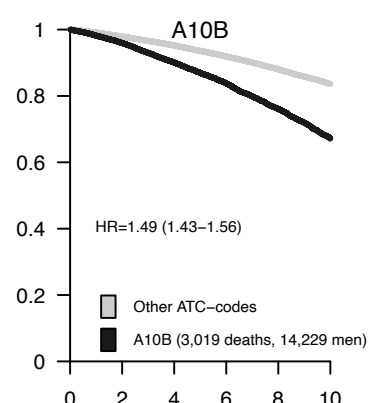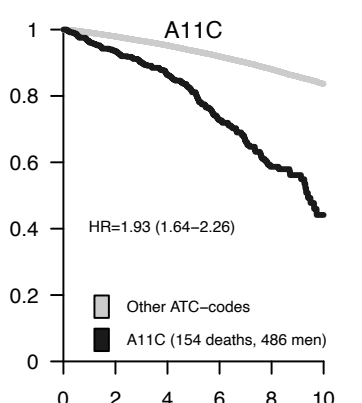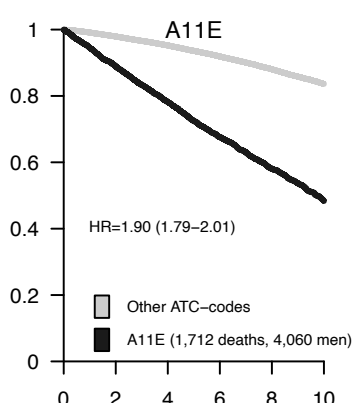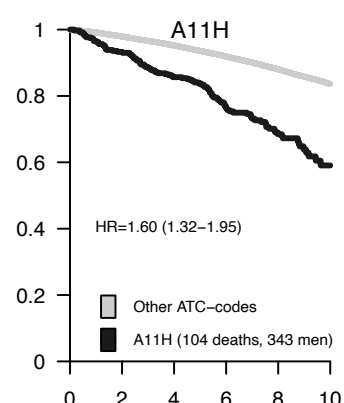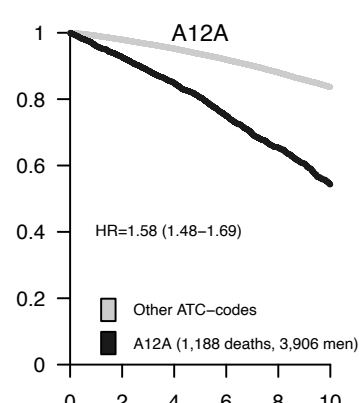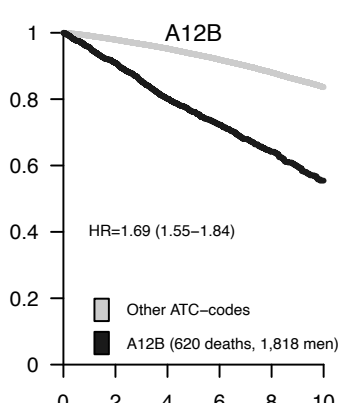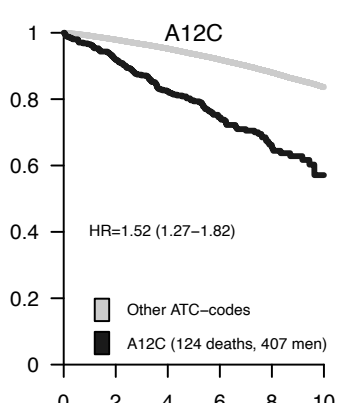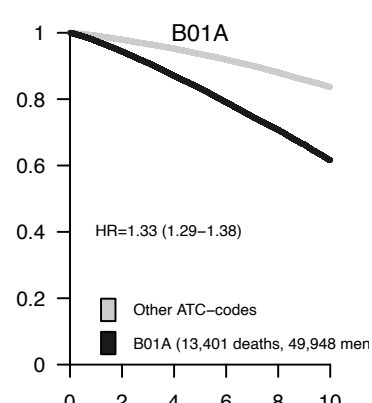

Supplement: S1 Fig — The number of events and total number of subjects in each category are presented in the legends. 95% confidence intervals (CI) are shown for the HRs. The analysis has been restricted to men with CCI = 0. (PDF) [file pone.0241439.s002.pdf]

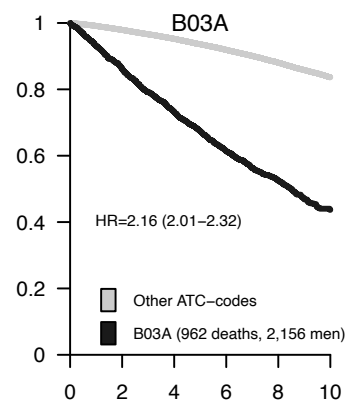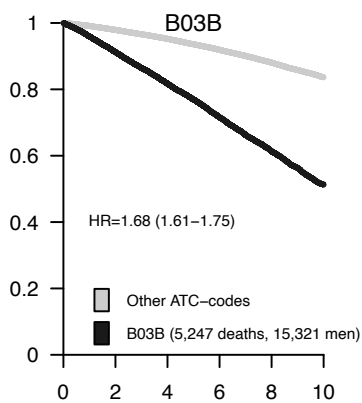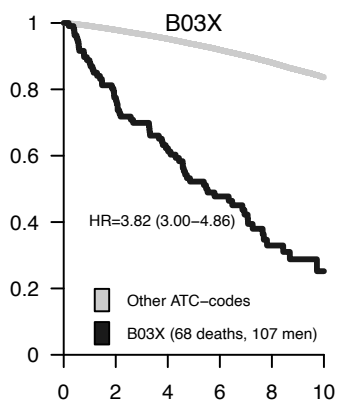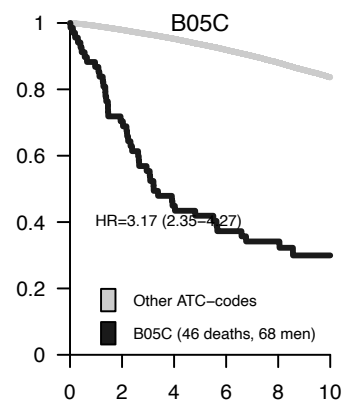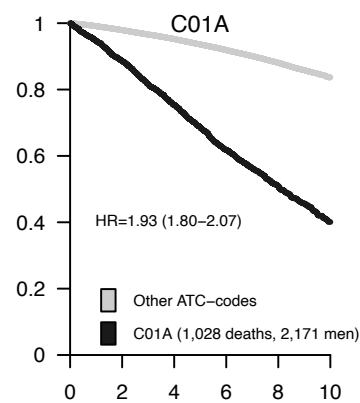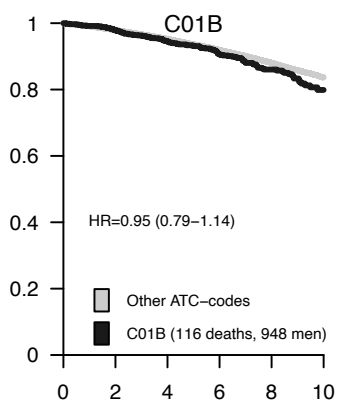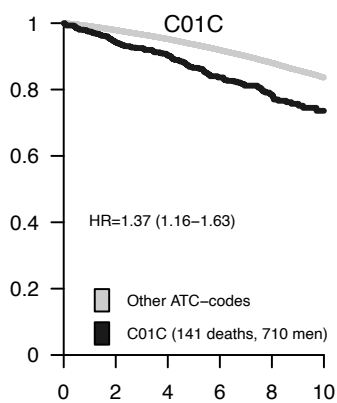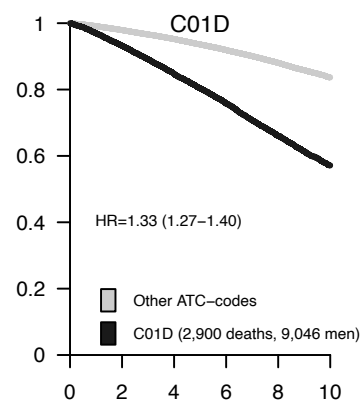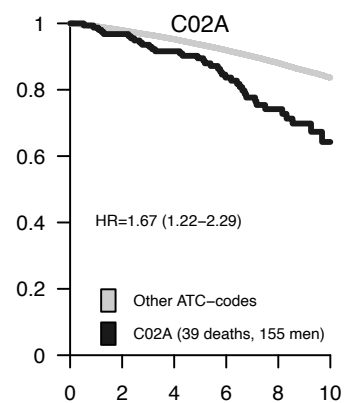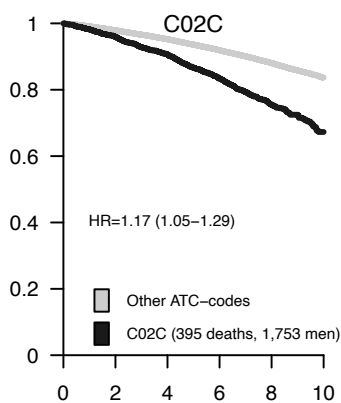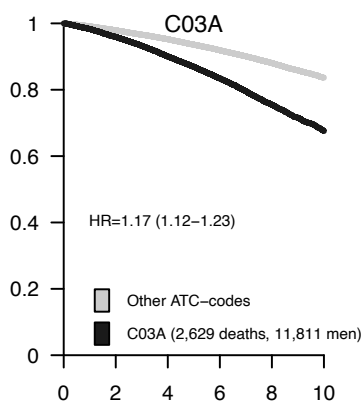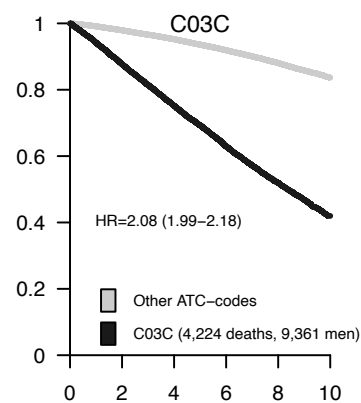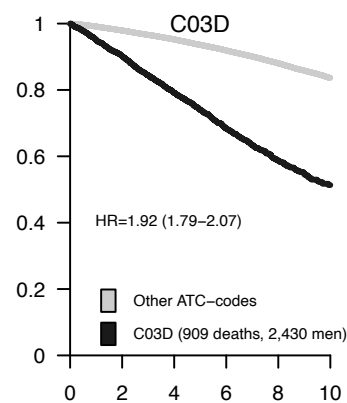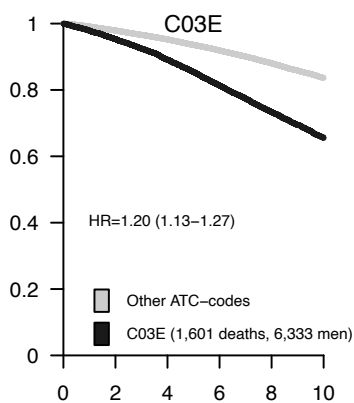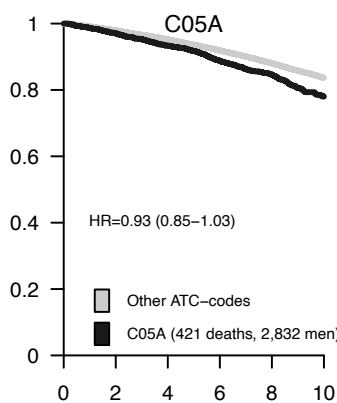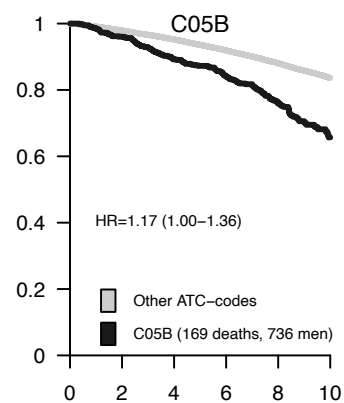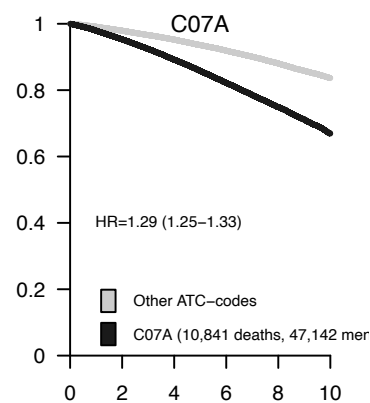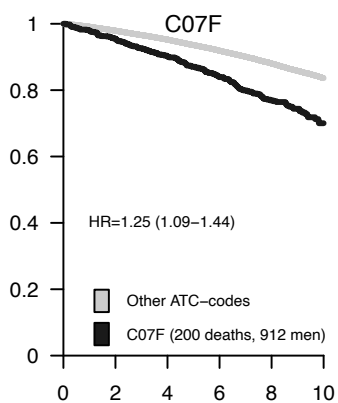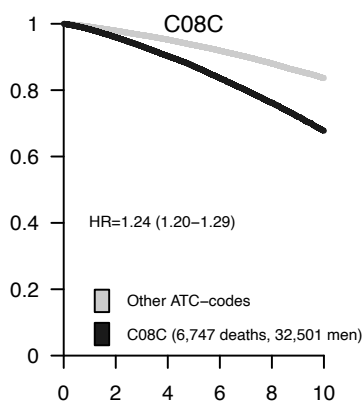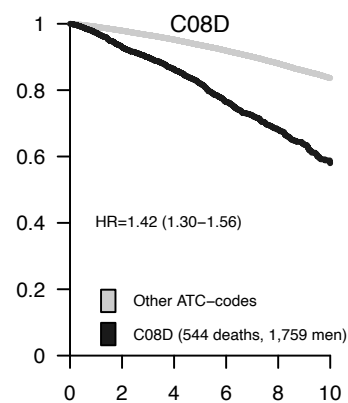

Supplement: S2 Fig — The number of events and total number of subjects in each category are presented in the legends. 95% confidence intervals (CI) are shown for the HRs. The analysis has been restricted to men with CCI = 0. (PDF) [file pone.0241439.s003.pdf]

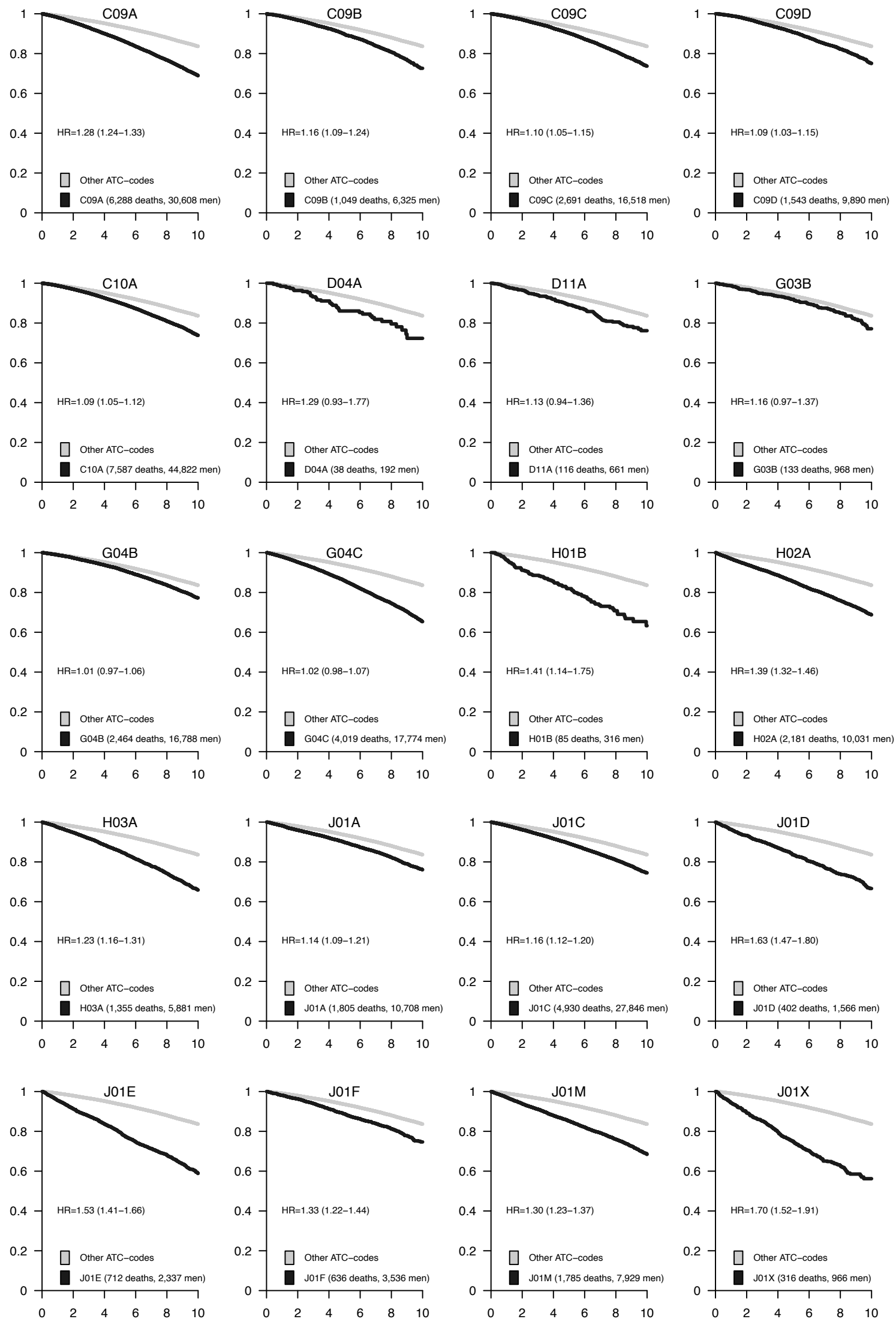

Supplement: S3 Fig — The number of events and total number of subjects in each category are presented in the legends. 95% confidence intervals (CI) are shown for the HRs. The analysis has been restricted to men with CCI = 0. (PDF) [file pone.0241439.s004.pdf]

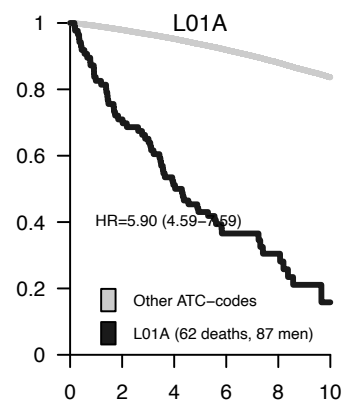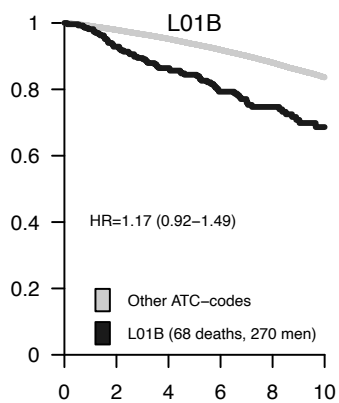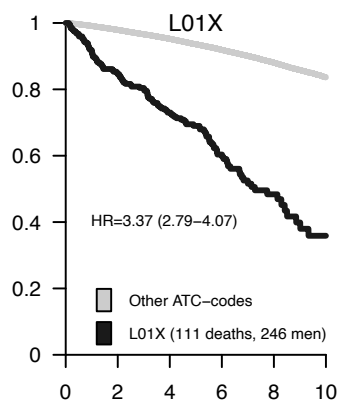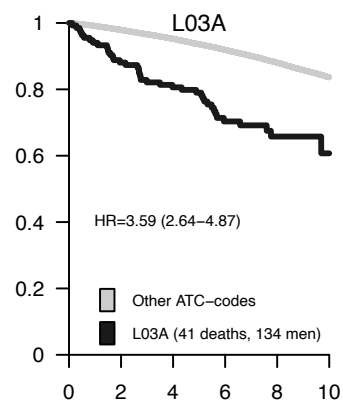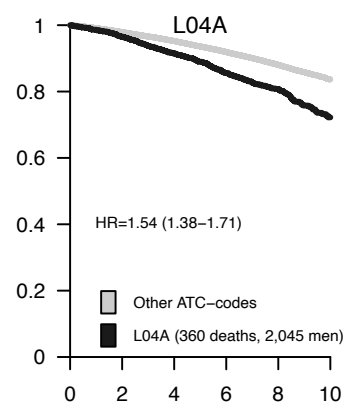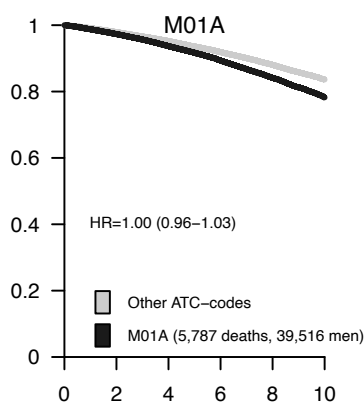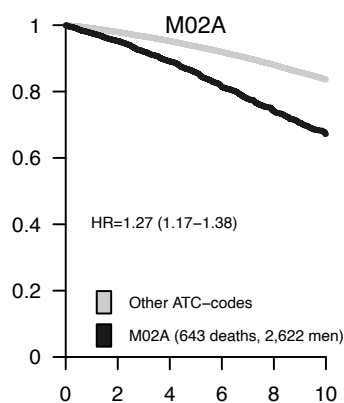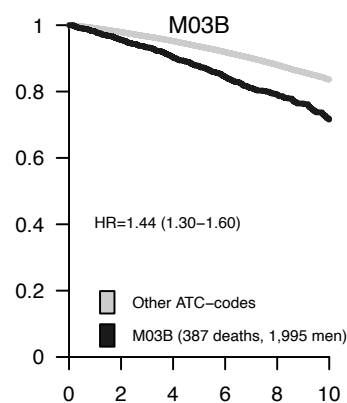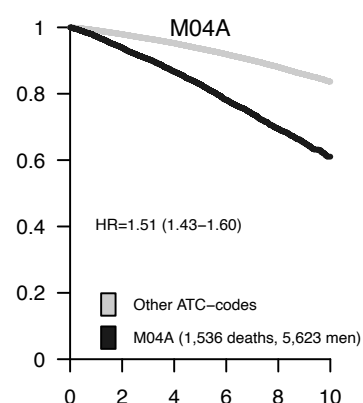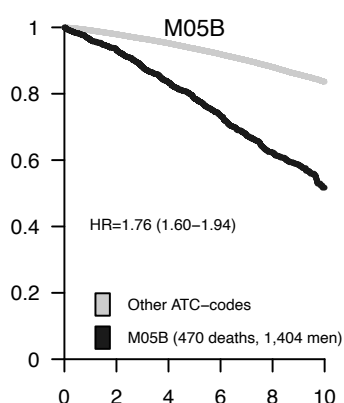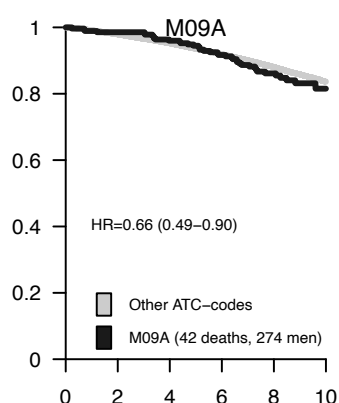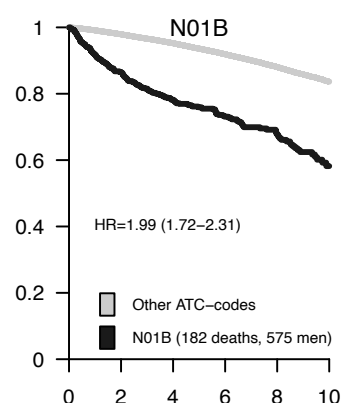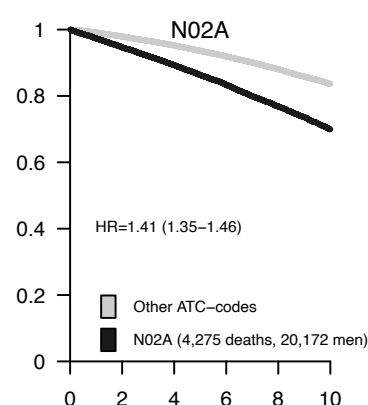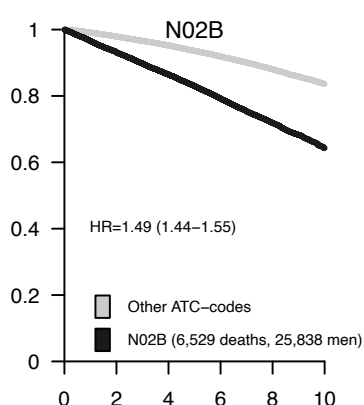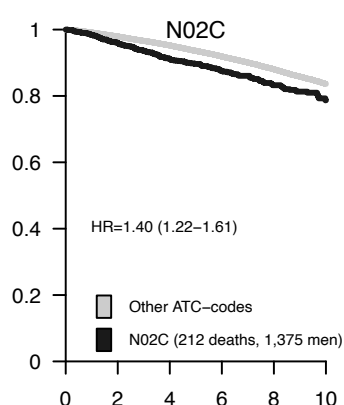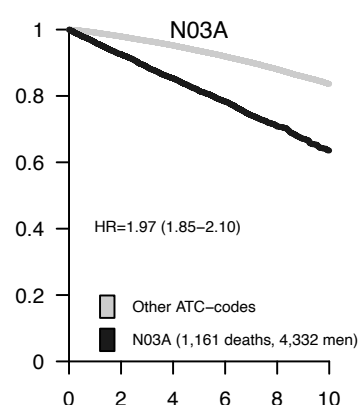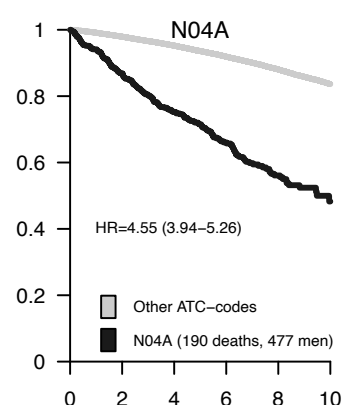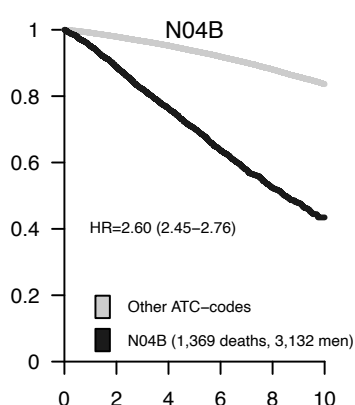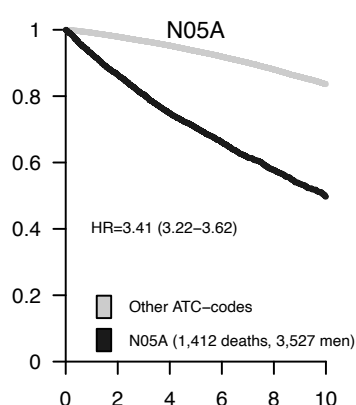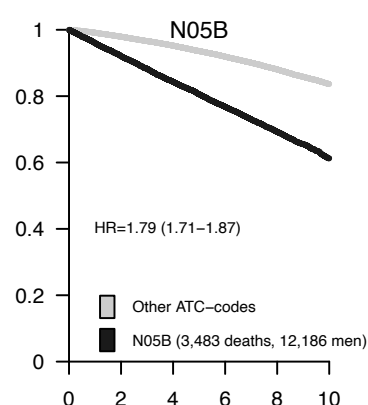

Supplement: S4 Fig — The number of events and total number of subjects in each category are presented in the legends. 95% confidence intervals (CI) are shown for the HRs. The analysis has been restricted to men with CCI = 0. (PDF) [file pone.0241439.s005.pdf]

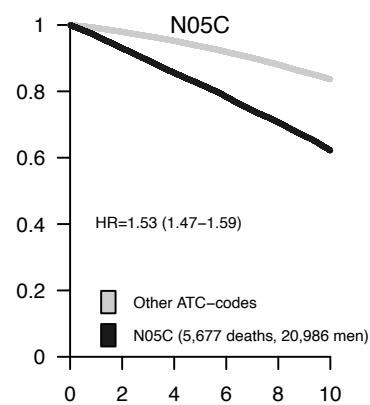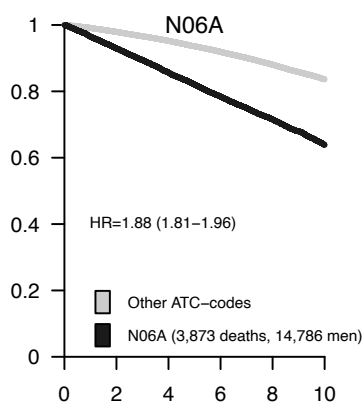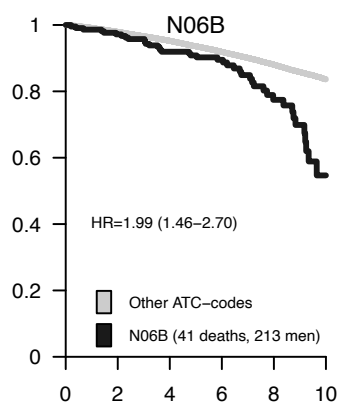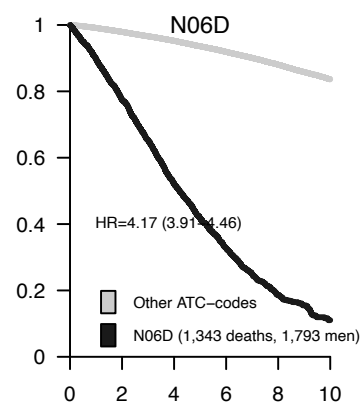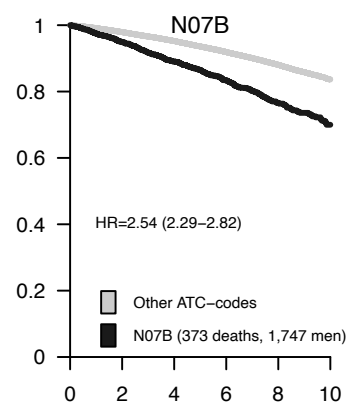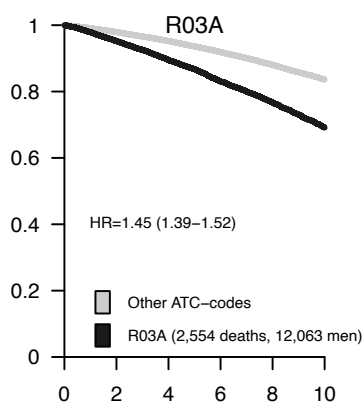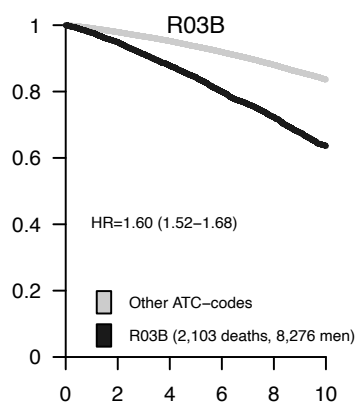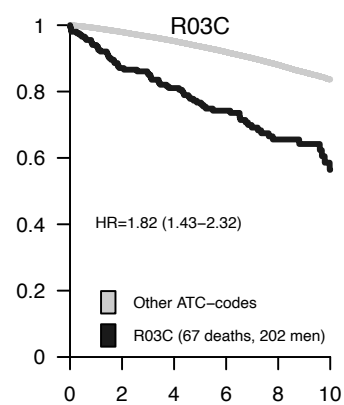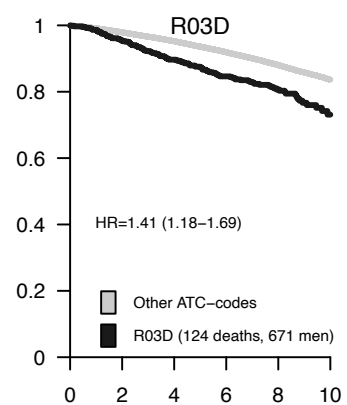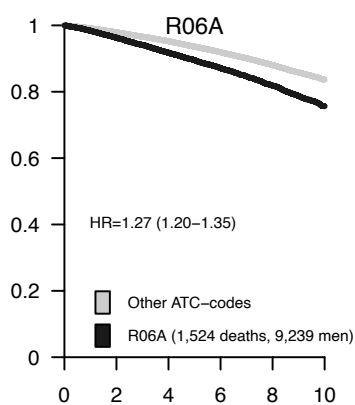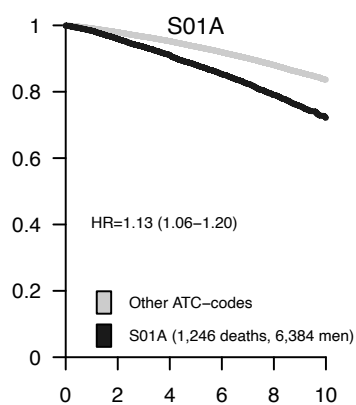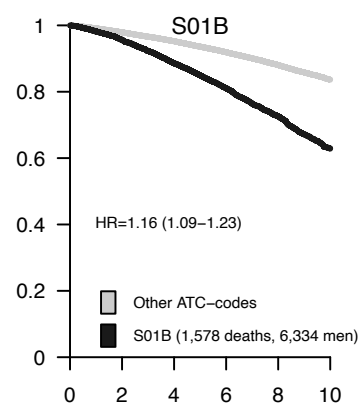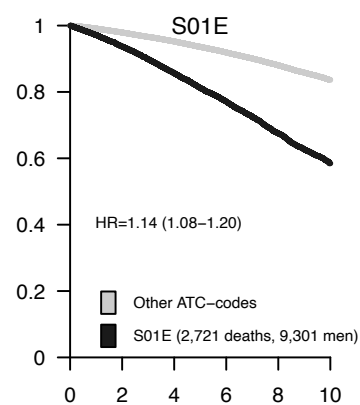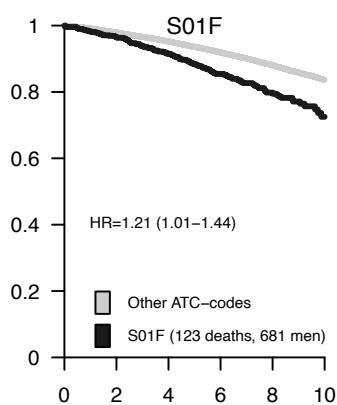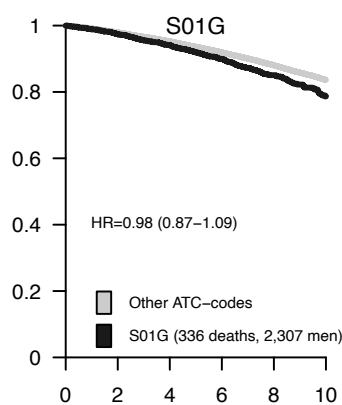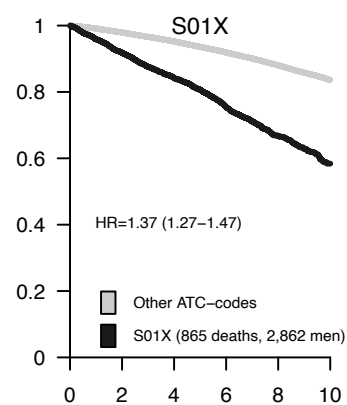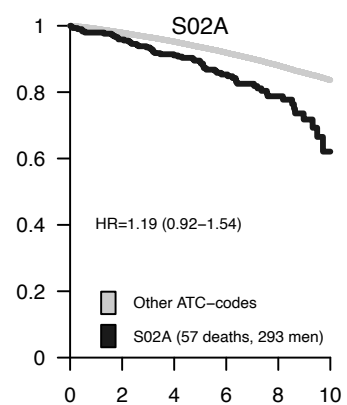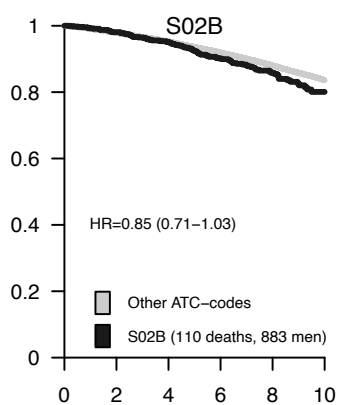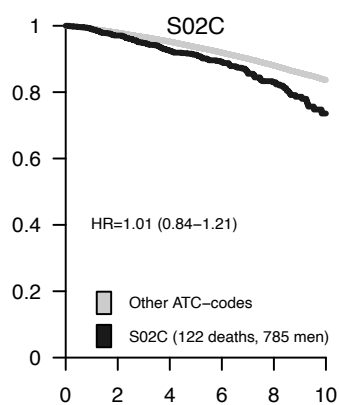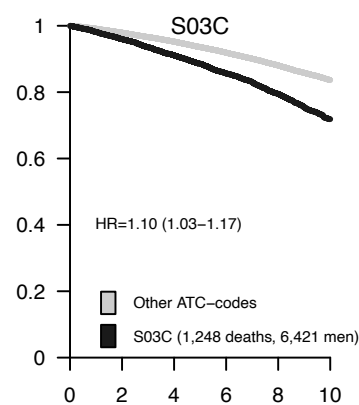

Supplement: S5 Fig — The number of events and total number of subjects in each category are presented in the legends. 95% confidence intervals (CI) are shown for the HRs. The analysis has been restricted to men with CCI = 0. (PDF) [file pone.0241439.s006.pdf]
