# Supplementary material for: Prescription-based prediction of baseline mortality risk among older men
Source: PLoS One. 2020 Oct 29;15(10):e0241439. doi: 10.1371/journal.pone.0241439 (PMC7595371; doi:10.1371/journal.pone.0241439)
Supplement: Attachment — Submitted filename: Letter to the editor PONE revision 1 Sept 2020_final.docx [file pone.0241439.s007.docx]

18 September 20

Dear Editor,

Thank you for considering our manuscript for publication. We have thoroughly considered the Editor’s and the reviewer’s comments, accepted most suggestions and revised the manuscript accordingly, which we believe has improved the manuscript and provides a stronger support for the main conclusion of the study. The revision of the manuscript has also undergone a formal language review by Editage^®^.

The following revisions have been made:

## Editor’s comments:

1. I disagree that 16% of the population and indeed 52,677 individuals are too few to be the main comparison group. I would much prefer them to be used as the reference - it would certainly add consistency and general usefulness to the analyses. Please change the reference group.

   Authors’ response:

We fully understand the Editor’s argument. To meet this request, we now in the main analysis use individuals without any prescription during the year before start of follow-up as the reference group. This has consistently increased the HR:s but not changed the relation between different ATC-codes. Se figure below (also provided as supplementary material):

We have, however, kept the original analysis as a sensitivity analysis, for reasons summarized below.

- It was the natural choice to have individuals without any prescription as the reference group. When realizing that this group only comprised 16% of the study population this raised our concern. It means that having no history of a prescription is not the expected or “normal” in this population of mainly elderly men. This is instead a characteristic of a minority. They can be expected to have unique, but unknown, characteristics, such as being especially healthy, having a very healthy life style, or for various reasons avoiding seeking medical care in situations where most people would seek medical attention. We have also added an assessment of baseline characteristics for men who had not filled any prescription. We found that they were younger, were more often married, and had a lower educational level (new supplemental table S2).
- The ultimate goal of the present study was to illustrate that prescriptions belonging to an ATC-code provide relevant information regarding the patient’s health status even if the indication is unknown or the use of the drug is not associated with mortality risk per se. The information on prescribed medications and the difference in strength of the association between each individual drug and risk of death is therefore of key interest.

Revision of the manuscript:
All estimated associations and accompanying confidence intervals in the results section in both the abstract and the main text have changed, and for some also the narrative description of the strength of the association. Please see tracked version of manuscript.

2. The methods section does not describe in detail in what time window during the study the prescriptions were made. Using “anytime during follow-up” would load massive amounts of immortal-time bias - especially in that you now use “individuals with at least one prescription” as the reference group. Please state how you defined prescription in terms of time and make sure all prescriptions preceded study entry. (Preferably all prescriptions filed the year (or two years) before study entry – this would also improve the usefulness of the methods in other epidemiological studies). Or alternatively in a time-variant manner (even though I believe most researchers would not use that in research practice for adjustment purposes).

   Authors’ response:
   Fully agree and this has been clarified in the manuscript.

   Revision of the manuscript:
   Page 5, row 82 (Methods):
   “Filled prescriptions were extracted **during the 1-year period preceding each individual’s start of follow-up**.”
3. The discussion lacks a discussion of why you did not perform the study in a population including women and potential lack of generalizability due to this fact.

   Authors’ response:
   Fully agree and this has been clarified in the manuscript.

Revision of the manuscript:
P17, line 329 (Discussion):
“For example, there was an association between use of iron supplement and mortality rate in our study, suggesting existence of indications for iron supplementation that are otherwise associated with increased risk of mortality in men. However, in a population of younger women, there could be indications for iron supplementation that are not associated with increased risk of mortality, such as excessive menstruation bleeding. Another example is use of antiandrogens (G03H) that could be expected to be associated with mortality, but not in a population selected as free of prostate cancer at baseline. **Since our research focus is on the epidemiology of prostate cancer our study population was restricted to males and essentially excluded men with prostate cancer, which is a notable limitation of our study. These observations of potential important influence of how the population was selected suggest that a population- and context-specific model might be warranted when using this type information to estimate baseline risk in practice. This should be one focus for future research in this area.**”

4. Please add analyses of “any drug prescription” as suggested by reviewer 1.

   Authors’ response:
   Added as requested.

   Revision of the manuscript:
   Page 6, row 130 (Results):

“While 73% were categorized as free of comorbidity according to the CCI, 84% had at least 1 prescription filled during the year before the start of follow-up. **These men tended to be older and have lower educational status, compared to the minority not having any prescription during the year before start of follow-up. Having at least one prescription was associated with a 60% increased mortality rate (HR = 1.60, 95% CI 1.56 to 1.64), compared to not having any prescription**.”

5. The discussion of furosemide. You need to add that the association is likely due to the drug being commonly prescribed in “palliative cancer, heart failure etc” patients to relieve swollen legs and pulmonary edema. It is therefore not at all surprising that it is associated to poor survival.

   Authors’ response:
   The discussion has been expanded as suggested.

   Revision of the manuscript:
   In our dataset, loop diuretics and potassium-sparing agents were strongly associated with mortality rate, while thiazide diuretics, and diuretics combined with potassium-sparing agents had weaker associations. **Within the drug class diuretics there is consequently substantial differences between different substances. This is expected since loop diuretics are prescribed in association with more severe conditions, such as heart and kidney failure, compared to thiazide diuretics. This underlines the importance of** information carried in the third level ATC code, specifically, the pharmacological subgroup, over the information carried by the first level code, i.e. anatomical main group.
6. I think your findings on cardiovascular drugs is because indeed individuals with hypertension only tend to end-up in this ATC-group and they may be very healthy and health conscious. It may also be an artefact of using the “some other drug” reference group. Please discuss, there are several references on claims data and hypertension on this topic.

   Authors’ response:
   While the change of reference group increased HR:s there was no change in relation to other drugs. We do not fully agree that e.g. class I and III antiarrhythmics and cardiac stimulants would be mainly prescribed to patients with hypertension. We have still expanded the discussion on this topic as requested. We would, however, like to emphasize that our main point is that while there are associations that are unexpected and difficult to explain, no such explanation is necessary for the information to be used as predictive of baseline mortality risk. However, note that such evaluation was not within the aim of the present study.

*We have subsequently used the results from this study to develop an aggregated comorbidity measure and we have compared the discriminative accuracy of this measure with other predictors, such as age, the Charlson comorbidity index, and another prescription-based index. The study has been submitted to another journal and is currently under review*.

Revision of the manuscript:
Page 17, row 560 (Discussion):

“…Most major cardiovascular conditions were expected to be associated with mortality. **While some drugs in this category are commonly prescribed for hypertension and therefore not expected to be strongly associated with mortality rate, unexpectedly** weak associations were seen for class I and III antiarrhythmics, cardiac stimulants (excluding cardiac glucosides; C01C), and vasodilators (including e.g. nitrates). Speculatively, prescribers are aware of risks associated with the use of e.g. antiarrhythmics, and therefore use of these drugs is largely avoided in frail patients.”

7. Consider using age as a continuous and quadratic in your analyses - it is the strongest known predictor of mortality.

   Authors’ response:
   We agree with the reviewer that age is a strong predictor of baseline mortality risk. It’s also known that the lifetime hazard of death follows a ‘bathtub’ curve. As Cox-regression is based on a proportional hazards assumption we decided to run a stratified Cox-regression where the stratification was based on age. This was done in order to meet the proportional hazards assumption.

We have also analyzed all models without stratification and instead adjusted for age as a continuous and quadratic term in the Cox model. The results are very similar. The HR for the eight first ATC-codes in Figure 1 are presented here for your information. We think this justifies our approach.

| **ATC-code** | **Stratified on age** | **Adjusted for age** |
| --- | --- | --- |
| A02A | 4.74 (4.40−5.09) | 4.57 (4.26-4.92) |
| A02B | 2.06 (2.00−2.12) | 2.02 (1.96-2.08) |
| A03A | 1.99 (1.87−2.12) | 1.93 (1.81-2.06) |
| A03B | 2.39 (1.98−2.87) | 2.41 (2.00-2.89) |
| A03F | 4.66 (4.38−4.96) | 4.53 (4.26-4.81) |
| A04A | 10.25 (9.42−11.14) | 10.17 (9.36-11.06) |
| A06A | 2.78 (2.70−2.87) | 2.72 (2.64-2.81) |
| A07A | 3.81 (3.56−4.08) | 3.73 (3.49-3.99) |

Revision of the manuscript:
Page 5, row 113 (Methods, Statistics):

**“…In a subgroup analysis we restricted the population to men with CCI = 0. Stratification for age was used instead of adjustment for age as a continuous variable, to avoid violation of the assumption of proportional hazards. We therefore also performed a sensitivity analysis without stratification and instead adjusted for age as a continuous and quadratic term in the Cox model.”**

Page 13, row 531 (Results):

**“…Adjusting for age as a continuous and quadratic term in the Cox models instead of stratifying for age produced nearly identical hazard ratios (data not shown).”**

8. In table 1 CCI=0 the number (n=23,7515) should be changed to 237,515.

   Authors’ response:
   Thank you for noting this error. Revised as suggested.

## Reviewer #1:

*Abstract*

1) The authors want to offer a method that is better than Charlson to predict mortality.
   If so it would be nice to see:
   a) prescription data limited to patients with Charlson = 0. What does prescription data add there?

      Authors’ response:
      A subgroup analysis restricted to CCI = 0 is reported in the results section with figures provided in the supplemental material.

      Revision of the manuscript:
      N/A
   b) and a model where both Charlson index + Prescriptions are added, how much does the prescription data add then?

      Authors’ response:
      This is clearly an interesting question, but not within the scope of the present study. Based on the findings in the present study we have performed a subsequent study where we present an integrated comorbidity measure based on prescription data. We compared this measure with age, the Charlson comorbidity index and another prescription-based comorbidity index. The subsequent study has been submitted for publication to another journal.

      Revision of the manuscript:
      N/A
2) could the men in question have other types of cancers than prostate cancers?

   Authors’ response:
   The men in the study population were selected as controls from the general population to men with prostate cancer. This means that they did not have prostate cancer on the index date when they were selected. This limitation has been clarified in the discussion (please see response to Editor’s question 3 above). They could, however, have any other cancer or later be diagnosed with a prostate cancer.

   Revision of the manuscript:
   Please see response to Editor’s question 3 above.

*Introduction*

3) While this may be the first study to explore mortality according to ”all prescribed drugs” it cannot possible be the first to describe ”all prescription” and comorbidity. I know that many authors use” number of drugs prescribed last 12 months” as a covariate for their comorbidity research. Gedeborg et al should acknowledge that and mention that in their paper.

Authors’ response:
We acknowledge the comment and have revised the introduction accordingly.

Revision of the manuscript:
**The association between different aspects of prescription history and mortality has been comprehensively evaluated in several previous studies [3, 4].** Prescription history as a measure of comorbidity has also been described previously [5]. A common approach has been to take a number of relevant comorbidities as the starting point and identify the Anatomical Therapeutic Chemical (ATC) codes for drugs used to treat these conditions [6]. This may, however, inadvertently overlook important information regarding the patients’ baseline mortality risk, suggesting a need for an approach involving non-specific prescriptions. **This study aimed to broadly explore prescription history, i.e. including all available prescribed drugs, to describe their potential usefulness for characterization of baseline mortality risk.**

*Methods*

4) Did the research ethics review really approve the study protocol? you had a detailed study protocol?

   Authors’ response:
   The comment is acknowledged and the text has been revised accordingly.

   Revision of the manuscript:
   The study ~~protocol~~ was approved by the Research Ethics Board in Uppsala that waived the informed consent requirement.
5) Please describe the Prescribed Drug Register with a few words (+ when it began), and what drugs are not registered? and that inpatient drugs are not covered.

   Authors’ response:
   The text has been revised to accommodate a response to the request.

   Revision of the manuscript:
   The National Prescribed Drug Registry is compulsory, nationwide, and run by the Swedish Board of Health and Welfare [10]. **It contains detailed and comprehensive information on all prescribed and dispensed drugs in Sweden, and includes the unique person identity number of the patient since 1 July 2005. It does not have information on drugs administered in the hospital setting.**
6) You counted the number of prescriptions. is that the most natural way to look at comorbidity? does ot dosage play a role.

   Authors’ response:
   Thank you for this interesting comment. The number of prescriptions could be one way to derive an aggregated measure of comorbidity. The development of such an aggregated measure is a natural continuation of research, based on the results we present in this study. But this is not within of the scope of the current study. We have already finalized such a study and it has been submitted to another journal for publication.
   The proposal to use number of prescriptions can be elaborated by entering each drug as a variable in a multivariable regression model. This way not only the count is used, but also a weight is attributed to each drug. Please note that this conceptually is an expansion of using the count, by using dummy-variables. But again, these analyses are not within the scope of the present manuscript.

   Revision of the manuscript:
   N/A

*Results*

7) It would be natural to present the overall HR for any prescription and risk of death.
   1 drug
   2 drugs
   3 drugs
   4 drugs or more…

   Authors’ response:
   Agree, please see the response to the question above.

   Revision of the manuscript:
   N/A

*Discussion.*

8) The association between several of the drugs and mortality, is ot strange considering what these drugs are prescribed for, add a few sentences on that

Authors’ response:
Agreed, and the text has been expanded accordingly.

Revision of the manuscript:
Page 15, row 599 (Discussion):
Many ATC-codes **are found to be associated with mortality, as expected from the severity of the conditions for which they are indicated.**

Page 16, row 632 (Discussion):

In principle, there might be different reasons behind an association between a class of prescribed drugs and mortality. The first consideration is if the medication in itself is causally related to the estimated mortality risk. However, more likely, the prescription indicates a patient’s health condition, which impacts mortality risk. In other words, it is the indication and not the drug itself that affects the risk of death.

9) Please note both erythropoietin and atipropulsives are sometimes used as part of cancer management (that is when I use it myself.

   Authors’ response:
   Noted.

   Revision of the manuscript:
   Page 17, row 657 (Discussion):
   In the present study, symptomatic treatments without a strictly defined disease indication were strongly associated with mortality rate, likely reflecting poor general health. Nausea and vomiting are not life-threatening per se, but when prescription medication is needed to alleviate such symptoms, it might signal associated conditions, such as malignancy and associated treatment, or general frailty.
10) The long discussion (results) of different pharma categoreies is fine.
    I miss a discussion about. residual confoundig, add text, and also comment more on the limitations of this paper.

    Authors’ response:
    Noted. We are, however, not in full agreement with the Reviewer on the meaning of residual confounding in this context. We do not in any way claim that the association between the drug prescription and mortality rate is causal. Merely that it is predictive of mortality rate based on correlation. Residual confounding is therefore not an issue. The limitations section has been expanded as requested.

    Revision of the manuscript:
    Page 18, row 692 (Discussion – section on limitations):
    An important limitation of the prescription registry is that it does not cover medicines administered in-hospital. For example, cardiac stimulants (C01C) are used in severe cardiac failure and are likely associated with mortality rate. However, these drugs were not associated with mortality rate in this study, likely due to the fact that these drugs are only administered intravenously to hospitalized patients, and hence not recorded in the Swedish prescribed drug registry.
    The results from this study might not be directly generalizable to other settings since prescription patterns vary between countries and regions, and over time. The information carried by prescriptions might also to some extent vary depending on the population studied. In the present study, the population of elderly men free of prostate cancer represented a specific subset of the general population. For example, there was an association between use of iron and mortality rate in our study, suggesting existence of indications for iron supplementation that are otherwise associated with increased risk of mortality in men. However, in a population of younger women, there could be indications for iron supplementation that are not associated with increased risk of mortality, such as menstruation. Another example is use of antiandrogens (G03H) that could be expected to be associated with mortality, but not in a population selected as free of prostate cancer at baseline. **Since our research focus is on the epidemiology of prostate cancer our study population was restricted to males and essentially excluded men with prostate cancer, which is a notable limitation for generalization of study results. These observations of potential important influence of how the population was selected suggest that a population- and context-specific model might be warranted when using this type information to estimate baseline risk in practice. This should be one focus for future research in this area.**
11) also add: I would like to see some more emphasis on "number of drugs" and comorbidity rather than just different types of dugs (ATC groups).
    HRs for death according to 0 drug [ref], 1 drug, 2 drugs, 3 drugs etc last year.
    Cause-specific death according to number of drugs.
    How much does the addition of one drug shorten the life expectancy, in a say 70-yr-old person? HRs for death according to 0 drug [ref], 1 drug, 2 drugs, 3 drugs etc last year.
    Cause-specific death according to number of drugs.
    How much does the addition of one drug shorten the life expectancy, in a say 70-yr-old person?

    Authors’ response:
    Life expectancy is the AUC under the complete survival curve. It requires very long follow-up. The analysis of life expectancy is complicated but clearly of interest. It must e.g. accommodate the fact that comorbidity (CCI and a potential aggregated prescription score) change over time. An aggregated prescription index may e.g. change because of discontinuation of medications. Change in life expectancy must therefore consider such dynamics.

While clearly of interest, an aggregated comorbidity measure based on the results in the present study is beyond the scope of the present study and as stated previously a manuscript on this issue have been submitted . This is also the case for calculations of life expectancy which is an implementation of such a measure in further analyses.

Revision of the manuscript:
N/A
